# Diversification and adaptive sequence evolution of *Caenorhabditis *lysozymes (Nematoda: Rhabditidae)

**DOI:** 10.1186/1471-2148-8-114

**Published:** 2008-04-19

**Authors:** Hinrich Schulenburg, Claudia Boehnisch

**Affiliations:** 1 Department of Animal Evolutionary Ecology, Zoological Institute, University of Tuebingen, Auf der Morgenstelle 28, 72076 Tuebingen, Germany

## Abstract

**Background:**

Lysozymes are important model enzymes in biomedical research with a ubiquitous taxonomic distribution ranging from phages up to plants and animals. Their main function appears to be defence against pathogens, although some of them have also been implicated in digestion. Whereas most organisms have only few lysozyme genes, nematodes of the genus *Caenorhabditis *possess a surprisingly large repertoire of up to 15 genes.

**Results:**

We used phylogenetic inference and sequence analysis tools to assess the evolution of lysozymes from three congeneric nematode species, *Caenorhabditis elegans*, *C. briggsae*, and *C. remanei*. Their lysozymes fall into three distinct clades, one belonging to the invertebrate-type and the other two to the protist-type lysozymes. Their diversification is characterised by (i) ancestral gene duplications preceding species separation followed by maintenance of genes, (ii) ancestral duplications followed by gene loss in some of the species, and (iii) recent duplications after divergence of species. Both ancestral and recent gene duplications are associated in several cases with signatures of adaptive sequence evolution, indicating that diversifying selection contributed to lysozyme differentiation. Current data strongly suggests that genetic diversity translates into functional diversity.

**Conclusion:**

Gene duplications are a major source of evolutionary innovation. Our analysis provides an evolutionary framework for understanding the diversification of lysozymes through gene duplication and subsequent differentiation. This information is expected to be of major value in future analysis of lysozyme function and in studies of the dynamics of evolution by gene duplication.

## Background

Since their discovery by Ian Fleming, lysozymes have become an important model system in molecular biology, biochemistry, and structural biology with major biomedical importance [1]. They are ubiquitous enzymes known from almost all groups of organisms including phages, bacteria, protists, fungi, animals, and plants [2-7]. Several distinct lysozyme types are recognised, including the chicken-type, goose-type, invertebrate-type, or amoeba lysozymes [2,7,8]. Because of their ability to break up peptidoglycan (an important component of bacterial cell walls) and their induced expression upon pathogen exposure, their original function was suggested to be defence against bacterial infections. At the same time, some lysozymes are involved in digestion. This function is found in vertebrate and insect taxa, which obtain nutrition from microorganisms involved in decomposing organic matter, e.g. the vertebrate foregut fermenters like ruminant artiodactyls, leaf-eating monkeys, the bird hoatzin, and the *Drosophila *and *Musca *flies [5,9-11].

Lysozymes have additionally become an important model in studies of molecular evolution. The origin of a digestive function in the leaf-eating monkeys was found to show the characteristic signature of adaptive sequence evolution, i.e. the non-synonymous substitution rate was significantly larger than the synonymous substitution rate, strongly indicating that amino acid-changing mutations were favoured by natural selection [12,13]. Gene duplication appears to play an important role in lysozyme evolution. Impressive examples include the ruminant artiodactyls with at least seven genes per genome [10], *Drosophila *fruitlies with at least eleven loci [5,14], and the mosquito *Anopheles gambiae *with at least nine lysozymes [15]. In these examples, some lysozymes have a digestive function. Functional diversification is further indicated by variation in gene expression pattern (e.g., timing, tissue, expression level) and several biochemical characteristics. For instance, the digestive lysozymes differ from the antimicrobial lysozymes by an increased expression in the gut, their resistance to protease degradation, an acidic isoelectric point and pH optimum [5,9]. Taken together, these patterns are consistent with the specific role of gene duplication as a source of evolutionary innovation [16], as known for diverse gene families like the animal hox and the vertebrate MHC genes [17,18].

An unexpected diversity of lysozymes is found in nematodes of the genus *Caenorhabditis*. They contain up to 15 different lysozymes of two distinct types [19,20]: the invertebrate-type and another distinct type that is characterized by lysozymes from various protist taxa (hereafter termed protist-type lysozymes). Although the exact function of these enzymes has not as yet been assessed systematically, some of them are involved in pathogen defence [19-21]. In the current paper, we provide a framework for understanding diversification of the *Caenorhabditis *lysozymes. In particular, we explore the lysozyme genealogy and test the hypothesis that gene duplications associate with diversifying selection, as expected for a role in immunity against the usually rapidly evolving repertoire of pathogens. Lysozyme sequences are considered from the three *Caenorhabditis *species with completely sequenced genomes, i.e. *C. elegans*, *C. briggsae*, and *C. remanei *[22]. Their genealogies are reconstructed at both protein and DNA sequence level with the help of maximum likelihood (ML) tree inference methods [23]. Signatures of positive selection are assessed across branches of the inferred genealogy and across the aligned sequences with the help of the maximum likelihood approach developed by Ziheng Yang and co-workers [24,25]. The results are related to the current data on lysozyme function.

## Results

### Overview and general phylogenetic position of the *Caenorhabditis *lysozymes

**Figure 1:**
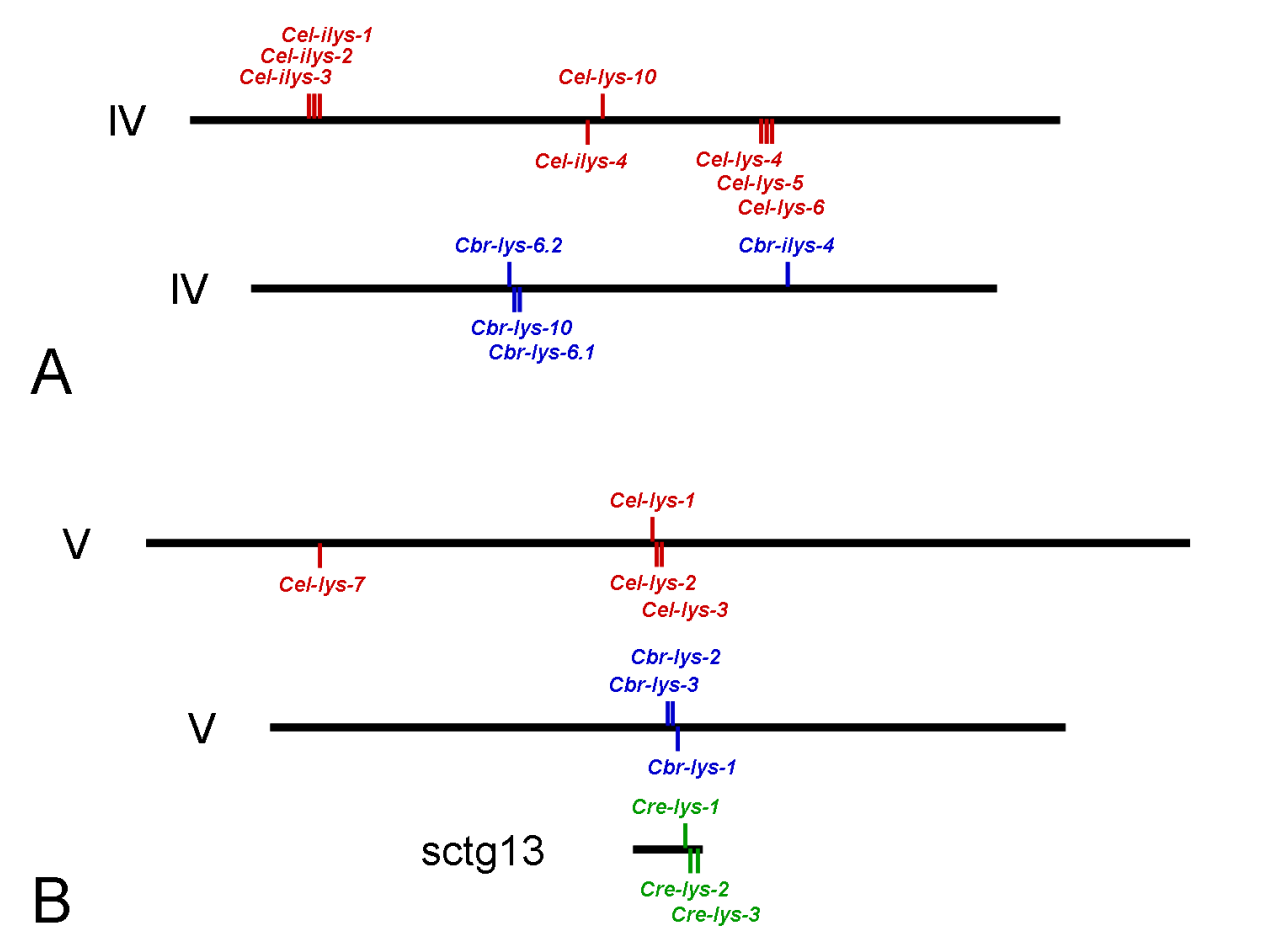
**Genomic distribution of the lysozyme genes on *A*, chromosome IV of *C. elegans *and *C. briggsae*, and *B*, chromosome V of *C. elegans *and *C. briggsae *and supercontig (sctg) 13 of *C. remanei***. Chromosomes of *C. elegans *and *C. briggsae *are drawn in proportion to their lengths. Position of genes is indicated by vertical lines, whereby lines above chromosomes indicate gene transcription from the sense strand and lines below chromosome transcription from the complementary strand.

**Figure 2:**
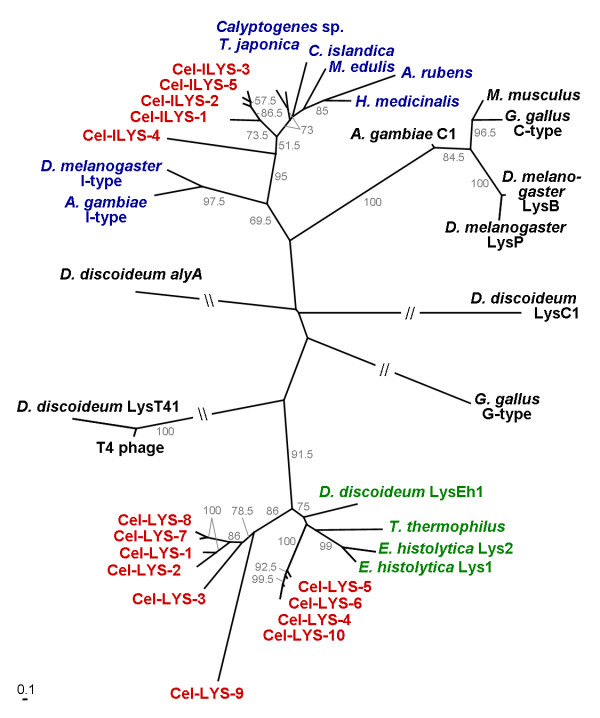
**Phylogenetic relationships between the *C. elegans *lysozymes (red labels) and the invertebrate-type (blue labels), protist-type (green labels), and also c-type, g-type, and phage-type lysozymes (all black labels)**. The tree was reconstructed from amino acid sequences using maximum likelihood. Branches are drawn in proportion to the inferred number of substitutions per site (see bar in bottom left corner). Bootstrap support from 200 replicate data sets is indicated next to branches. Only values larger than 50 are given. Branches interrupted by two slashes were shortened. The unrooted topology is shown, since the position of a possible root is unknown.

The lysozymes from the three *Caenorhabditis *species are listed in Table 1 and 2. The genomic distribution of clustered genes is illustrated in Fig. 1. As a first step, we compared all complete lysozyme protein sequences from *C. elegans *with those from various vertebrates, invertebrates, protists, and one phage. For this purpose, a multiple sequence alignment was generated based on a hierarchical method, i.e. similar sequences are aligned first, followed by alignment of less similar sequences (see methods). We noted that the resulting alignment almost exclusively contained variable positions. Moreover, if we varied the settings of the alignment algorithm (e.g. gap opening, gap extension, or gap distance penalties), only few regions could be recovered in identical form. Therefore, positional homology may not be entirely reliable. Since the alignment was inferred from the hierarchical algorithm, it should still be informative as to the general phylogenetic position of the nematode lysozymes. In fact, its phylogenetic analysis highlighted that the nematode possesses two distinct lysozyme types (Fig. 2; inferred from the alignment obtained using default settings of the alignment programme), thus confirming previous observations. In particular, five *C. elegans *lysozymes group with the invertebrate-type lysozymes. These lysozyme genes are thus labelled *Cel-ilys-1 *up to *Cel-ilys-5*. The remaining ten *C. elegans *lysozymes are the previously labelled genes *lys-1 *up to *lys-10*. They fall into two separate lineages within the distinct clade of protist lysozymes.

**Table 1:** Information on the invertebrate-type lysozymes

Species	Gene	Gene name/Protein ID	Chromosome	Prot. length	MW	pI	Charge	Hydropathy
*C. elegans*	*Cel-ilys-1*	C45G7.1/CE17548	IV	145	16.4	6.3	-2	-0.603
	*Cel-ilys-2*	C45G7.2/CE17549	IV	139	15.1	7.9	2	-0.228
	*Cel-ilys-3*	C45G7.3/CE24850	IV	139	15.0	8.1	3	-0.238
	*Cel-ilys-4*	C55F2.2/CE31458	IV	159	18.0	6.1	-2	-0.301
	*Cel-ilys-5*	F22A3.6/CE04442	X	139	15.1	8.1	3	-0.180

*C. briggsae*	*Cbr-ilys-4*	CBG17700/CBP19059	IV	241	27.6	9.0	11	-0.428
	*Cbr-ilys-5*	CBG10836/CBP02633	X	139	15.1	7.9	2	-0.222

*C. remanei*	*Cre-ilys-4.1*	cr01.sctg14.wum.205.1	sctg14	158	17.9	5.7	-2	-0.279
	*Cre-ilys-4.2*	cr01.sctg556.wum.3.1	sctg556	158	18.0	5.9	-2	-0.318
	*Cre-ilys-5*	cr01.sctg0.wum.479.1	sctg0	139	15.1	7.9	2	-0.174

Location of *C. remanei *genes can only be attributed to supercontigs (sctg). MW, molecular weight, to be multiplied by 1000. Isoelectric point (pI), charge, and grand average of hydropathy were calculated with the help of the ProtParam tool of the ExPASy server.

**Table 2:** Information on the protist-type lysozymes

Species	Gene	Gene name/Protein ID	Chromosome	Prot. length	MW	pI	Charge	Hydropathy
*C. elegans*	*Cel-lys-1*	Y22F5A.4/CE16605	V	298	32.4	5.5	-2	0.031
	*Cel-lys-2*	Y22F5A.5/CE16606	V	279	30.3	6.3	0	-0.039
	*Cel-lys-3*	Y22F5A.6/CE20201	V	301	33.8	5.4	-7	-0.156
	*Cel-lys-4*	F58B3.1/CE06003	IV	214	23.6	7.0	0	-0.045
	*Cel-lys-5*	F58B3.2/CE06004	IV	215	23.5	6.9	0	-0.001
	*Cel-lys-6*	F58B3.3/CE06005	IV	214	23.1	6.0	-2	0.168
	*Cel-lys-7*	C02A12.4/CE07828	V	283	30.9	6.7	0	0.048
	*Cel-lys-8*	C17G10.5/CE06846	II	286	31.0	5.8	-1	0.031
	*Cel-lys-9*	C54C8.6/CE08969	I	179	19.8	9.8	10	-0.251
	*Cel-lys-10*	F17E9.11/CE07076	IV	230	25.8	8.6	3	-0.140

*C. briggsae*	*Cbr-lys-1*	CBG09572/CBP08248	V	291	31.3	7.7	1	0.171
	*Cbr-lys-2*	CBG09573/CBP08249	V	276	30.0	6.3	0	-0.022
	*Cbr-lys-3*	CBG09574/CBP08250	V	300	33.9	5.2	-12	-0.132
	*Cbr-lys-6.1*	CBG06111/CBP07239	IV	214	23.1	7.7	1	0.133
	*Cbr-lys-6.2*	CBG06114/CBP21696	IV	216	23.5	6.9	0	0.014
	*Cbr-lys-8*	CBG02448/CBP00589	II	282	30.4	6.2	0	0.112
	*Cbr-lys-10*	CBG06112/CBP01531	IV	214	23.5	6.4	-2	-0.019

*C. remanei*	*Cre-lys-1*	cr01.sctg13.wum.291.1	sctg13	293	31.6	7.7	1	0.185
	*Cre-lys-2*	cr01.sctg13.wum.292.1	sctg13	288	31.1	6.6	0	0.092
	*Cre-lys-3*	cr01.sctg13.wum.297.1	sctg13	315	35.7	4.9	-12	-0.195
	*Cre-lys-6*	cr01.sctg3655.wum.3.1	sctg3655	214	23.2	7.7	1	0.070
	*Cre-lys-8.1*	cr01.sctg9.wum.10.1	sctg9	285	31.1	5.5	-1	-0.015
	*Cre-lys-8.2*	cr01.sctg9.wum.67.1	sctg9	282	30.7	6.6	0	0.037
	*Cre-lys-10*	cr01.sctg32.wum.160.1	sctg32	214	23.6	6.4	-2	-0.019

Location of *C. remanei *genes can only be attributed to supercontigs (sctg). MW, molecular weight, to be multiplied by 1000. Isoelectric point (pI), charge, and grand average of hydropathy were calculated with the help of the ProtParam tool of the ExPASy server.

For the more detailed phylogenetic analyses, we examined the two lysozyme types separately. For this purpose, we generated two new alignments and several subsets of these (see methods and below).

### Evolution of invertebrate-type lysozymes

**Figure 3:**
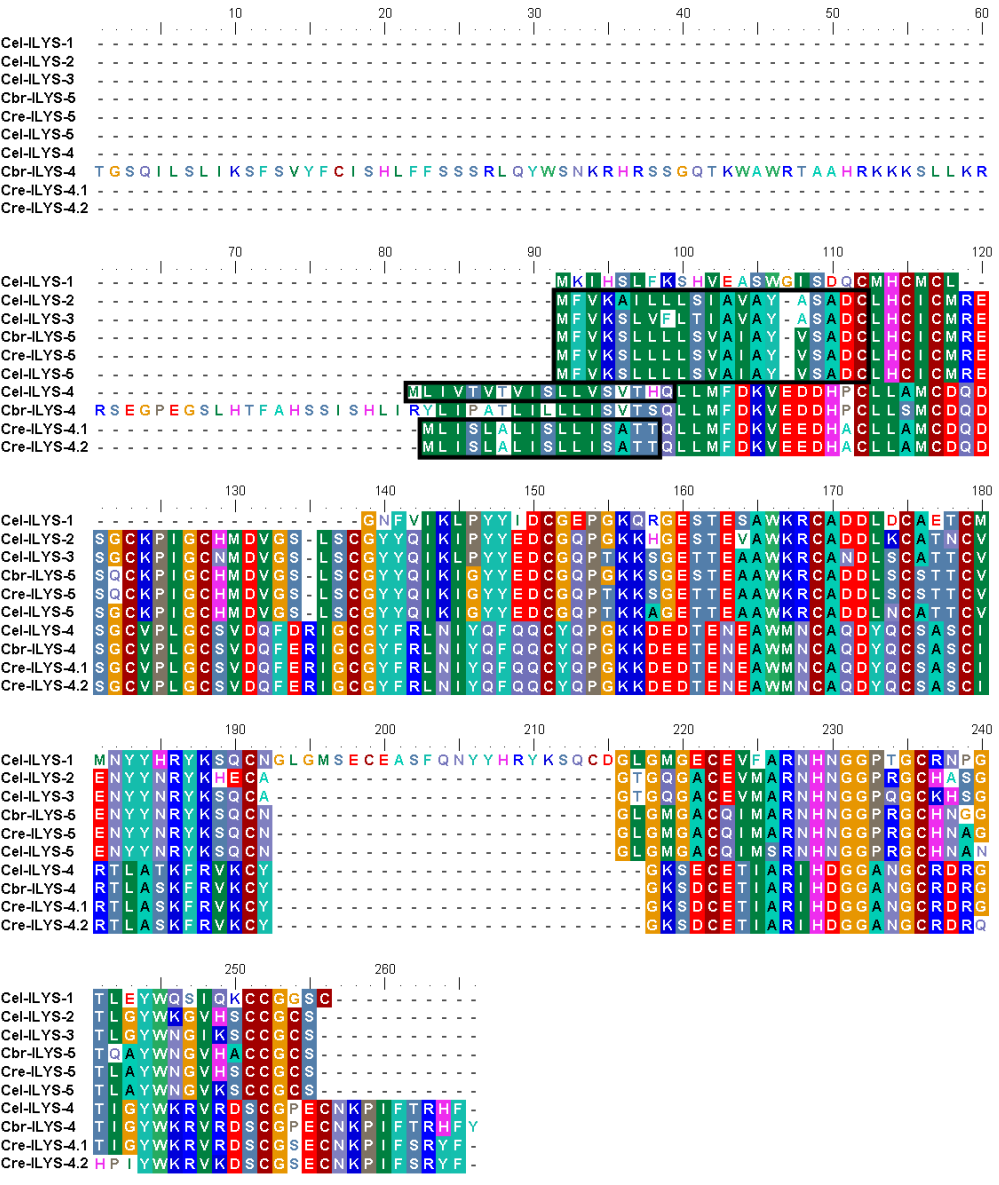
**Alignment of the *Caenorhabditis *invertebrate-type lysozyme amino acid sequences**. Black boxes indicate the inferred signal peptides.

The genomes of *C. briggsae *and *C. remanei *contain two and three invertebrate-type lysozyme genes, respectively. They were named in consideration of their similarity and phylogenetic affinity to the *C. elegans *lysozymes (see below; Table 1). Three of the five invertebrate-type lysozymes from *C. elegans *are found in a single cluster and with the same orientation on chromosome IV (Fig. 1A). None of the other genes are present in clusters (Table 1). All invertebrate-type lysozymes could be reliably aligned to each other at both protein (alignment 2; Fig. 3) and DNA sequence level (alignment 3; position of indels is identical between the two alignments). Two genes show unusual properties in comparison to the others and thus they may be non-functional (i.e. they are pseudogenes). In particular, the gene *Cbr-ilys-4 *possesses an unusual amino terminus and it lacks a signal peptide. *Cel-ilys-1 *contains a large insertion, it shows many nucleotide differences to the other sequences, and it also lacks a signal peptide.

**Figure 4:**
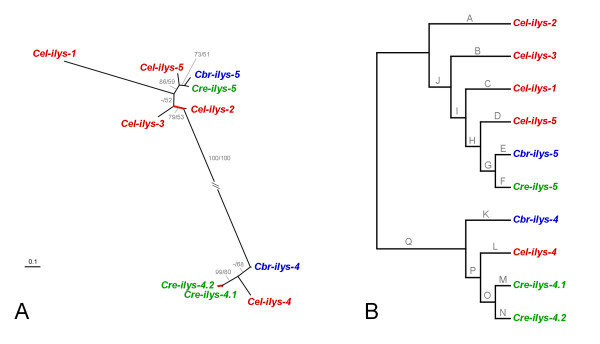
**Genealogy of the *Caenorhabditis *invertebrate-type lysozymes, including *A*, the unrooted tree topology with branch-lengths inferred from DNA sequence analysis, and *B*, the tree topology with branch-names used in the analysis of positive selection across branches**. The tree was inferred with maximum likelihood. In *A*, values before and after slashes refer to the bootstrap results inferred from protein and DNA sequence analysis, respectively. Only bootstrap values larger than 50 are shown. Branches in *A *are drawn in proportion to the estimated number of substitutions per site, as indicated by the bar in the bottom left corner. Red-coloured branches indicate those inferred to be under positive selection. The unrooted topology is the most appropriate representation of the genealogy since the exact position of the root is unknown. The representation in *B *serves to illustrate branch-names for the analysis of positive selection; the branch-names are identical to those given in Table 3.

Phylogenetic analysis of protein and DNA sequences yielded essentially identical tree topologies (Fig. 4A). The only two differences refer to (i) the exact position of *Cel-ilys-1*, *Cel-ilys-2*, and *Cel-ilys-3 *in relation to each other, and (ii) the position of *Cel-ilys-4 *and *Cbr-ilys-4 *in relation to the monophylum of *Cre-ilys-4.1 *and *Cre-ilys-4.2*. These discrepancies are reflected by low bootstrap support for the respective branches in both protein and DNA trees, indicating lack of sufficient unambiguous phylogenetic information in the sequences. Otherwise, the inferred genealogy identifies two distinct clades, one with the *ilys-4 *genes and the other with all remaining genes. Both clades contain genes from all three taxa.

Our analysis did not reveal any indication for adaptive sequence evolution across sequences (likelihood ratio test [LRT] comparison between model M8 with either model M7 or M8a, *P *≥ 0.5). However, we consistently identified two episodes of positive selection along the phylogeny, regardless of the analysis method (Table 3; Fig. 4). In both cases, adaptive sequence evolution associates with incidences of intra-lineage lysozyme radiations (in one case within the *C. remanei *and the other case within the *C. elegans *lineage; Fig. 4A). Most of the remaining branches have a *d*_*N*_/*d*_*S *_rate ratio well below 1, suggesting purifying selection (i.e. amino acid changes are selectively disfavoured).

**Table 3:** Results of the analysis of adaptive sequence evolution for individual branches of the invertebrate-type lysozyme tree.

Branch	Free-ratio		2-ratio		
		
	*d*_*N*_/*d*_*S*_	Bootstrap	*d*_*N*_/*d*_*S*_	2Δ*L*	*P*
A	0.089	74	3.883	2.942	0.0862
B	0.104	100	0.150	0.713	0.3985
C	0.105	100	0.097	0.004	0.9522
D	0.097	99	0.108	0.055	0.8140
E	0.076	100	0.063	0.293	0.5883
F	< 0.001	97	< 0.001	2.031	0.1541
G	0.212	79	0.229	0.642	0.4229
H	0.047	66	0.204	0.238	0.6258
I	1.454	33	0.219	0.531	0.4663
J	**> 999**	79	**> 999**	7.296	0.0069^#^
K	< 0.001	84	< 0.001	2.161	0.1416
L	0.041	100	0.038	2.794	0.0946
M	< 0.001	98	< 0.001	0.249	0.6180
N	**3.493**	87	**2.572**	7.653	0.0057*^,#^
O	0.056	100	0.044	1.831	0.1760
P	0.061	66	0.036	2.259	0.1329
Q	0.009	100	0.009	2.020	0.1552

Branch names are as depicted in Fig. 4B. For the first comparison, *d*_*N*_/*d*_*S *_rate ratios were inferred for individual branches with the free-ratio model, in which all branches were allowed to vary; the optimal model had a likelihood of ln *L *= -2871.29; the significance of individual branches having a *d*_*N*_/*d*_*S *_rate ratios above 1 or below 1 was assessed with non-parametric bootstrapping using 100 replicates; *d*_*N*_/*d*_*S *_rate ratios larger than 1 and with bootstrap support of more than 50 are given in bold. For the second comparison, *d*_*N*_/*d*_*S *_rate ratios were repeatedly inferred with the 2-ratio model, in which only the branch of interest was allowed to differ from the remaining branches; the significance of the individual branches to be different from the remaining branches was assessed via a likelihood ratio test comparison to the null model, in which all branches of the tree were assumed to have identical *d*_*N*_/*d*_*S *_rate ratios; the null model had a likelihood score of ln *L *= -2895.56; the probability *P *was calculated from twice the likelihood difference 2Δ*L *between null model and tested model; significance is indicated by * and ** for α = 0.1 and 0.05 according to the sequential Bonferroni procedure, respectively, and by ^# ^and ^## ^for α = 0.1 and 0.05 according to the false discovery rate, respectively; bold *d*_*N*_/*d*_*S *_rate ratios indicate those that are significantly larger than 1 according to either method.

### Evolution of protist-type lysozymes

The protist-type lysozymes are present with either seven (both *C. briggsae *and *C. remanei*) or ten genes (*C. elegans*; Table 2). Synteny is found for the genes *lys-1*, *lys-2*, and *lys-3*, which are clustered in all three species – in both *C. elegans *and *C. briggsae *on chromosome V and in *C. remanei *on supercontig 13 (Table 2; Fig. 1B). The gene *lys-1 *is always found in opposite orientation to the other two. In *C. remanei*, the *lys-3 *homologue is separated from the other two genes by approximately 10,000 nucleotides (and four open-reading frames) in contrast to both *C. elegans *and *C. briggsae*, where the three genes are directly adjacent to each other. In *C. elegans*, the *lys-7 *gene is additionally found on chromosome V, but in a different location than the three clustered genes (Fig. 1B). *C. elegans *contains a second well-defined cluster of protist-type lysozymes on chromosome IV, including *Cel-lys-4*, *Cel-lys-5*, and *Cel-lys-6*. In this case, there is no synteny in the other species. Interestingly, however, the *C. briggsae *chromosome IV contains a cluster that combines genes from the above *C. elegans *cluster (in this case the *C. briggsae *genes *Cbr-lys-6.1 *and *Cbr-lys-6.2*) with the gene *Cbr-lys-10*. The *C. elegans *orthologue of the latter gene, *Cel-lys-10*, is similarly present on chromosome IV but in a different location than the cluster (Fig. 1A). In *C. remanei*, two additional genes are found in relatively close physical proximity to each other: *Cre-lys-8.1 *and *Cre-lys-8.2 *are located on supercontig 9 (Table 2) separated by approximately 200,000 nucleotides and 56 open-reading frames.

**Figure 5:**
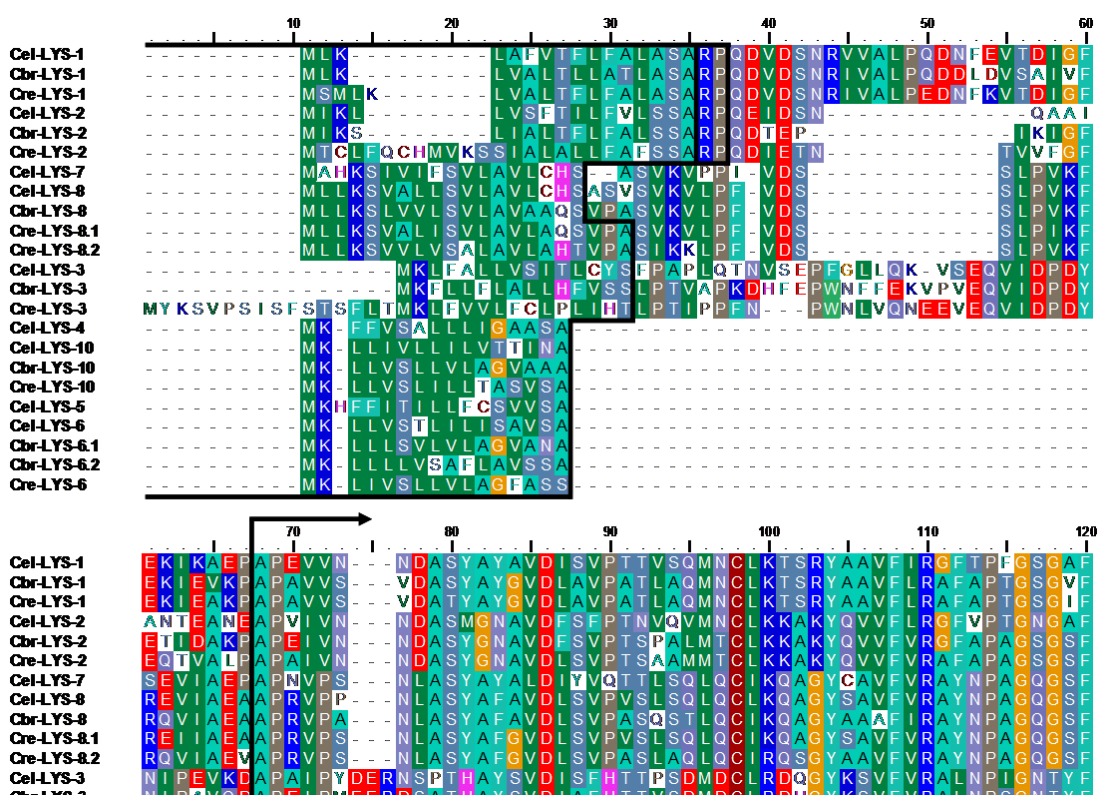
**Alignment of the *Caenorhabditis *protist-type lysozyme amino acid sequences**. The figure only shows the top quarter of the alignment. The complete alignment is given in Additional file 1. In both cases, black lines at the beginning of the alignment denote the inferred signal peptides. Alignment 4 (see methods and results) includes all taxa and the entire protein sequences. Vertical black lines with arrows below the alignment indicate the regions used for specific DNA sequence analysis of all protist-type lysozymes (alignment 5). Vertical black lines with arrows above the alignment indicate those regions analyzed for the clade 1 lysozymes (alignments 6 and 7 for protein and DNA sequences, respectively). Clade 2 lysozyme analysis was based on complete sequences (alignments 8 and 9 for protein and DNA sequences, respectively). Note that all alignments are subsets of alignment 4, i.e. the position of indels is identical. The red box and arrow indicate the sequence position, which was inferred to be under positive selection for the clade 1 lysozymes.

The overall phylogeny of the protist-type lysozymes from nematodes and one outgroup taxon (*Dictyestelium discoideum*) was assessed with an alignment of the complete protein sequences (alignment 4; Fig. 5 and Additional file 1). This alignment was robust to variations of the settings of the alignment programme. In contrast, for the corresponding DNA sequences, several regions could not be recovered in identical form under similar variations. Therefore, it cannot be entirely excluded that these regions bear an increased risk of homoplasy. To reduce this risk for the detailed analysis of lysozyme evolution (i.e. inference of non-synonymous and synonymous substitution rates), we extracted five subsets from alignment 4. Of these, alignment 5 consists of the alignable part of all protist-type lysozyme DNA sequences from the Caenorhabditis nematodes (see vertical black lines with arrows below the alignment in Fig. 5/Additional file 1). Since alignment 5 considered only a comparatively short part of the genes, we additionally analyzed the clade 1 and 2 protist-type lysozymes separately. These separate analyses allowed us to include complete or almost complete genes and thus additional phylogenetic information as contained in the regions excluded in alignment 5. Here, analysis of clade 1 lysozymes was based on the alignable part of the genes (see vertical black lines with arrows above alignment in Fig. 5/Additional file 1; alignments 6 and 7 for protein and DNA sequences, respectively), while that of the clade 2 lysozymes included the complete protein or DNA sequences (alignments 8 and 9, respectively; Fig. 5/Additional file 1). We would like to emphasize that alignments 5–9 are subsets of alignment 4 as indicated in Fig. 5 and Additional file 1 (i.e. position of indels is identical between alignments). *Cel-lys-9 *was always excluded because it did not permit reliable alignment to the other lysozymes.

**Figure 6:**
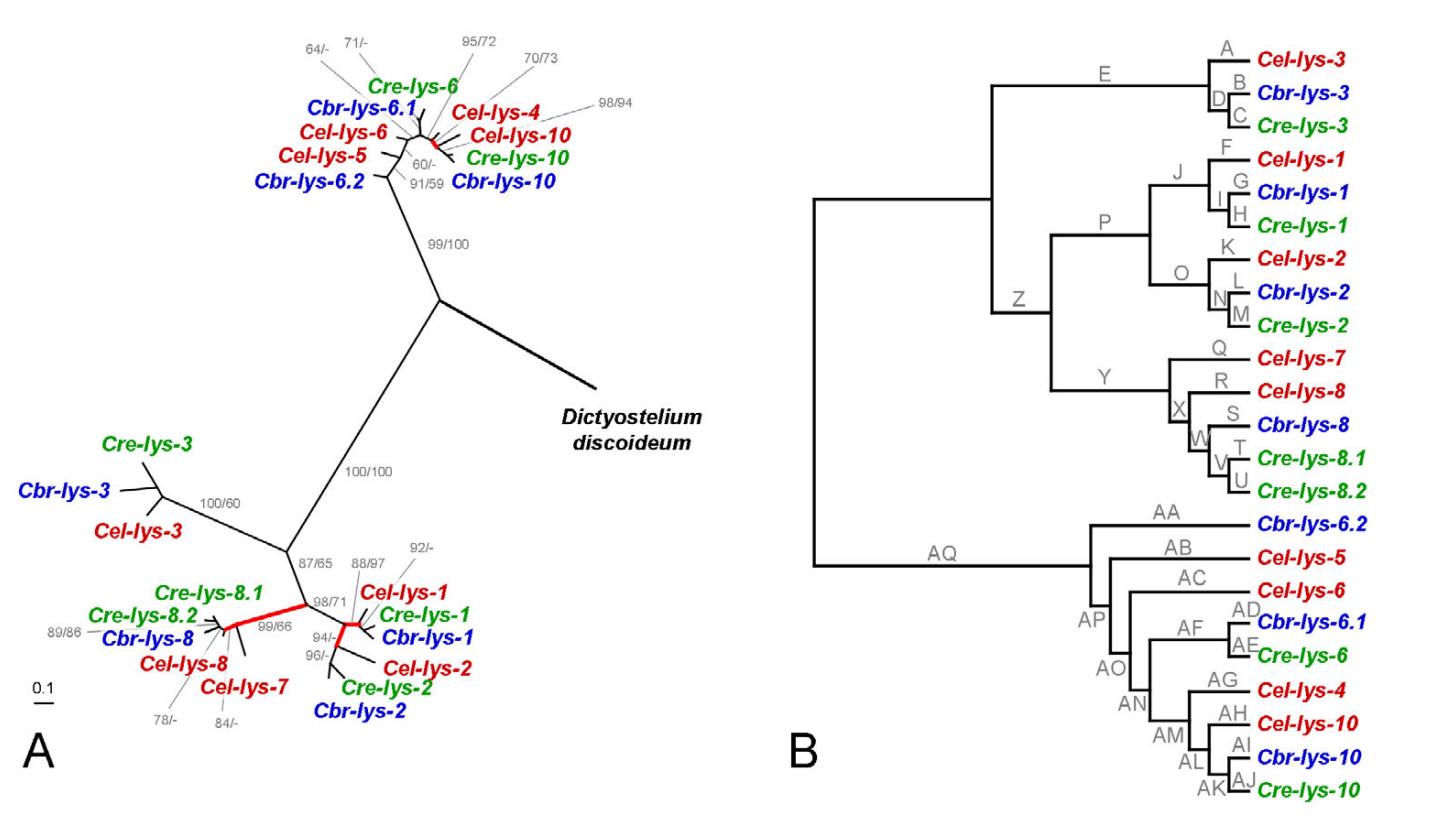
**Genealogy of all *Caenorhabditis *protist-type lysozymes, including *A*, the unrooted tree topology with branch-lengths inferred from protein sequence analysis, and *B*, the tree topology with branch-names used in the analysis of positive selection across branches**. The branch-names in *B *are identical to those given in Table 4. All other information as in Fig. 4.

**Figure 7:**
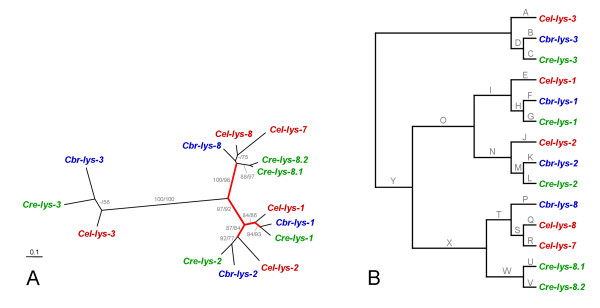
**Genealogy of the protist-type clade 1 lysozymes, including *A*, the unrooted tree topology with branch-lengths inferred from DNA sequence analysis, and *B*, the tree topology with branch-names used in the analysis of positive selection across branches**. The branch-names in *B *are identical to those given in Table 5. All other information as in Fig. 4.

**Figure 8:**
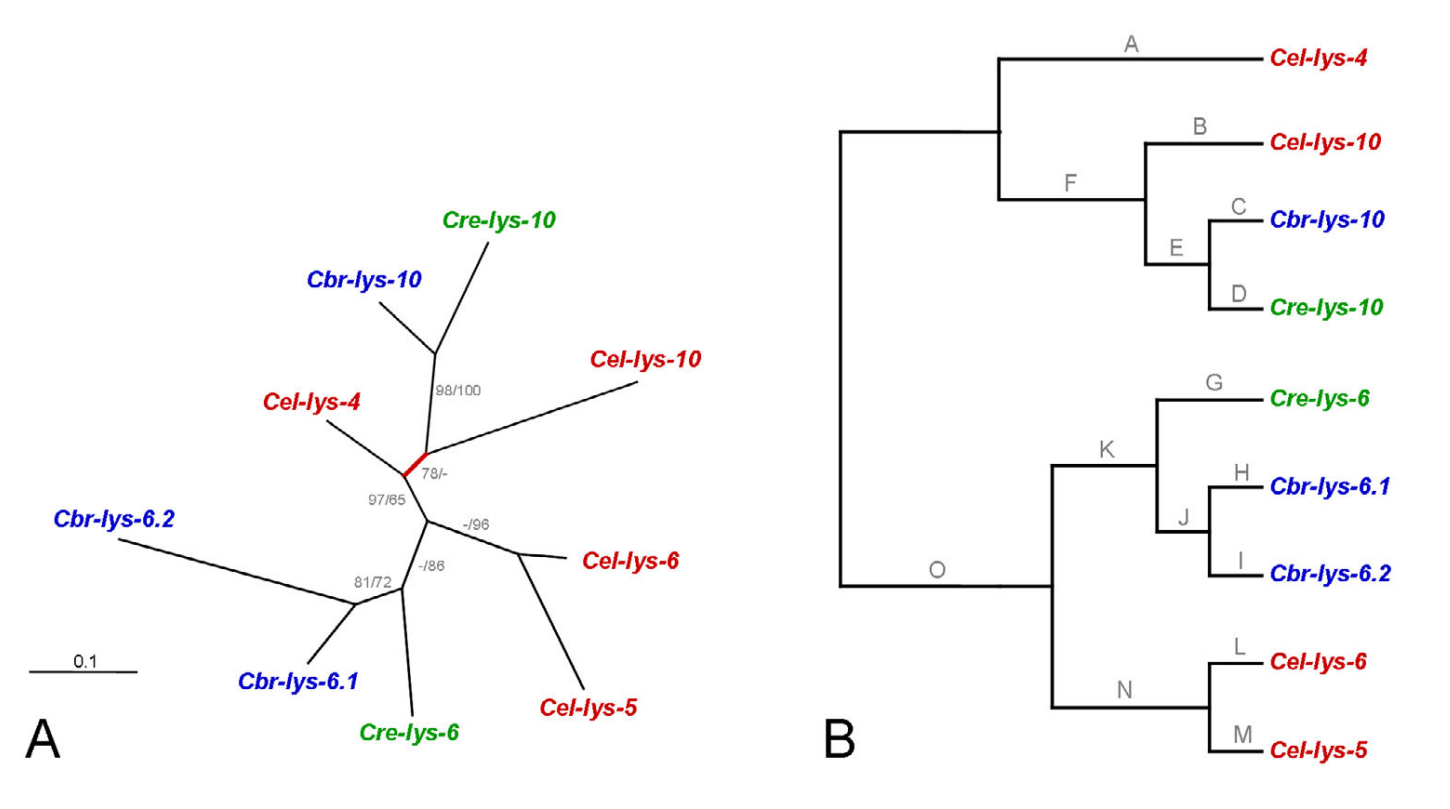
**Genealogy of the protist-type clade 2 lysozymes, including *A*, the unrooted tree topology with branch-lengths inferred from DNA sequence analysis, and *B*, the tree topology with branch-names used in the analysis of positive selection across branches**. The branch-names in *B *are identical to those given in Table 6. All other information as in Fig. 4.

For all data sets, protein and DNA sequence alignments yielded essentially identical tree topologies. The only differences referred to (i) the exact position of *Cel-lys-7*, *Cel-lys-8*, *Cbr-lys-8*, and the clade containing *Cre-lys-8.1 *and *Cre-lys-8.2 *in relation to each other (alignments 4–7; Figs. 6 and 7), and (ii) the exact position of *Cel-lys-5*, *Cel-lys-6*, *Cbr-lys-6.1*, *Cbr-lys-6.2*, and *Cre-lys-6 *in relation to each other (alignments 4, 5, 8, 9; Fig. 6 and 8). Almost all of these differences are again associated with low bootstrap support, suggesting that the available sequences lack sufficient unambiguous phylogenetic information at these two levels. All other relationships were consistently identified, irrespective of the alignment used, indicating the availability of robust phylogenetic information in these cases. The phylogenetic analysis yielded the following information.

(i) The protist-type lysozymes fall into two distinct clades (clade 1 and 2), which diverged before separation of the three species (Fig. 6A).

(ii) Within clade 1, four distinct phylogenetic groups are identified (Fig. 7A). Three of them contain one orthologue per species, indicating duplication of genes before species separation. The fourth group includes one gene for *C. briggsae*, two monophyletic genes for *C. elegans*, and two monophyletic genes for *C. remanei*.

(iii) The inferred clade 2 topology shows less hierarchical structure than the clade 1 topology (Fig. 8A). Here, the *lys-10 *orthologues form a monophyletic group, which is most closely related to *Cel-lys-4*. The remainder of this clade shows a single gene from *C. remanei*, two monophyletic genes from *C. elegans*, and two monophyletic genes from *C. briggsae*.

The analysis of positive selection across sequence alignments yielded a single significant result. In particular, for the aligned clade 1 coding sequences (alignment 7, see methods and Fig. 5) model M8 differed significantly from both model 7 (LRT, 2ΔL = 8.786, P = 0.012) and model 8a (LRT, 2ΔL = 6.589, adjusted P = 0.005). A single alignment position was found to be subject to adaptive sequence evolution according to the Bayes empirical Bayes method (P = 0.99). The alignment position is found in the middle of the genes and it is highlighted in Fig. 5. For the other data sets, the comparisons were all insignificant

During assessment of adaptive sequence evolution along branches, the different data sets and the two methods for inference of statistical significance produced slightly different results. For instance for the clade 1 sequences, only a single branch was inferred to have a *d*_*N*_/*d*_*S *_rate ratio significantly above 1 by both methods (the branch leading to the *lys-1 *orthologues). In the analysis of the complete data set (including both clade 1 and 2), the same branch was found to be significant by only one of the two approaches. In spite of these variations, the results from all data sets and methods, taken together, consistently point to two main tree regions that are likely to be subject to positive selection: (i) the branch leading to the *lys-10 *orthologues (Tables 4 and 6; Figs. 6 and 8), and (ii) the branches associated with the early radiation of the *lys-1*, *lys-2*, and *lys-7/8 *orthologues (Tables 4 and 5; Figs. 6 and 7). The majority of the remaining branches yielded a *d*_*N*_/*d*_*S *_rate ratio that was clearly below 1, indicating purifying selection.

**Table 4:** Results of the analysis of adaptive sequence evolution for individual branches of the whole protist-type lysozyme tree.

Branch	Free-ratio		2-ratio		
		
	*d*_*N*_/*d*_*S*_	Bootstrap	*d*_*N*_/*d*_*S*_	2Δ*L*	*P*
A	0.024	91	0.021	3.167	0.0751
B	0.057	80	0.056	1.064	0.3024
C	0.080	96	0.049	1.550	0.2131
D	0.129	68	0.023	2.224	0.1359
E	0.365	61	0.002	1.746	0.1864
F	0.049	100	0.051	0.726	0.3942
G	0.013	100	< 0.001	7.951	0.0048^#^
H	0.022	100	0.018	3.961	0.0466
I	0.085	73	0.019	0.342	0.5588
J	0.853	41	**> 999**	11.627	0.0006**^,##^
K	0.094	100	0.116	0.087	0.7680
L	0.131	100	0.141	0.399	0.5278
M	0.112	100	0.172	0.412	0.5212
N	0.613	62	> 999	4.752	0.0293
O	**> 999**	97	> 999	6.263	0.0123
P	0.303	77	> 999	5.772	0.0163
Q	0.177	100	0.237	2.696	0.1006
R	0.017	99	0.027	1.360	0.2435
S	0.087	100	0.077	0.146	0.7022
T	0.173	97	0.180	0.280	0.5964
U	0.177	90	0.214	0.447	0.5039
V	0.029	100	0.029	3.183	0.0744
W	0.171	85	0.073	0.100	0.7524
X	**3.860**	59	> 999	3.589	0.0582
Y	0.740	58	**> 999**	8.385	0.0038^#^
Z	2.148	47	17.722	1.898	0.1683
AA	0.016	93	0.027	0.665	0.4148
AB	0.451	54	8.852	1.815	0.1779
AC	0.072	98	0.137	0.050	0.8228
AD	0.074	100	0.056	0.836	0.3605
AE	0.027	100	0.025	2.859	0.0909
AF	0.091	95	0.102	0.000	0.9929
AG	0.073	100	0.106	0.005	0.9447
AH	0.046	100	0.055	1.221	0.2691
AI	0.039	100	0.024	2.779	0.0955
AJ	< 0.001	99	< 0.001	10.084	0.0015*^,##^
AK	0.236	95	0.287	1.944	0.1632
AL	**> 999**	90	> 999	4.708	0.0300
AM	0.366	70	0.581	2.098	0.1475
AN	0.266	94	0.361	3.621	0.0570
AO	0.382	71	0.982	1.682	0.1947
AP	0.188	81	0.080	0.241	0.6235
AQ	0.713	53	> 999	3.275	0.0703

Branch names are as in Fig. 6B. Methods and abbreviations as in Table 3. The optimal free-ratio model had a likelihood of ln *L *= -5556.07 and the 1-ratio null model for LRT comparison had ln *L *= -5646.46.

**Table 5:** Results of the analysis of adaptive sequence evolution for individual branches of the clade 1 protist-type lysozyme tree.

Branch	Free-ratio		2-ratio		
	*d*_*N*_/*d*_*S*_	Bootstrap	*d*_*N*_/*d*_*S*_	2Δ*L*	*P*

A	0.050	92	0.044	1.965	0.1610
B	0.063	100	0.058	1.849	0.1739
C	0.053	100	0.043	4.309	0.0379
D	0.128	49	0.035	1.477	0.2242
E	0.089	100	0.143	0.203	0.6522
F	0.012	100	0.008	10.130	0.0015**^,##^
G	0.023	100	0.017	8.769	0.0031*^,##^
H	**24.042**	58	0.365	1.228	0.2678
I	**1.065**	62	**> 999**	8.981	0.0027*^,##^
J	0.132	100	0.182	1.614	0.2040
K	0.086	100	0.088	0.226	0.6345
L	0.099	100	0.098	0.025	0.8754
M	0.384	76	0.983	4.410	0.0357
N	0.312	85	**> 999**	8.148	0.0043*^,##^
O	0.555	66	**> 999**	8.984	0.0027*^,##^
P	0.116	100	0.096	0.066	0.7974
Q	0.028	100	0.028	3.446	0.0634
R	0.221	100	0.205	3.798	0.0513
S	0.100	86	0.059	0.384	0.5353
T	< 0.001	93	< 0.001	4.235	0.0396
U	0.109	97	0.104	0.001	0.9703
V	0.158	100	0.168	0.557	0.4557
W	0.047	100	0.034	4.648	0.0311
X	0.892	59	**2.616**	8.715	0.0032*^,##^
Y	0.351	93	0.482	3.526	0.0604

Branch names as in Fig. 7B. Methods and abbreviations as in Table 3. The optimal free-ratio model had a likelihood of ln *L *= -6048.14 and the 1-ratio null model for the LRT comparison had ln *L *= -6117.89.

**Table 6:** Results of the analysis of adaptive sequence evolution for individual branches of the clade 2 protist-type lysozyme tree.

Branch	Free-ratio		2-ratio		
		
	*d*_*N*_/*d*_*S*_	Bootstrap	*d*_*N*_/*d*_*S*_	2Δ*L*^c^	*P*^d^
A	0.112	100	0.128	0.013	0.9110
B	0.080	100	0.085	0.879	0.3483
C	0.085	100	0.058	1.548	0.2134
D	0.020	100	0.019	10.800	0.0010**^,##^
E	0.132	96	0.157	0.149	0.6996
F	**> 999**	83	> 999	3.322	0.0684
G	0.050	100	0.048	2.752	0.0972
H	0.063	100	0.062	0.903	0.3420
I	0.157	100	0.145	0.302	0.5825
J	0.079	97	0.047	1.152	0.2832
K	0.170	95	0.149	0.089	0.7652
L	0.088	100	0.147	0.065	0.7991
M	0.435	95	0.440	9.683	0.0019**^,##^
N	0.237	97	0.336	2.723	0.0989
O	0.482	59	1.088	2.572	0.1088

Branch names are as depicted in Fig. 8B. Methods and abbreviations as in Table 3. The optimal free-ratio model had a likelihood of ln *L *= -3525.65 and the 1-ratio null model for the LRT comparison had ln *L *= -3555.98.

### Characteristics and function of the different lysozymes

Tables 1 and 2 list the characteristics of *Caenorhabditis *lysozymes, highlighting variation in length, molecular weight, isoelectric point, charge, and the grand average hydropathy. Importantly, the three distinct clades differ significantly in all of these traits with the exception of charge (Table 7). The most pronounced differences are found between the clade 1 protist-type lysozymes and the invertebrate-type lysozymes (posthoc tests in Table 7). Although the two protist-type lysozyme clades are generally more similar to each other, they do show some variation, especially regarding length and weight.

**Table 7:** Differences in the characteristics of the three main lysozyme clades

Clades/Statistics	Prot. length	MW	pI	Charge	Hydropathy
Clades					
1: p-lys clade 1	289.93 ± 2.83	31.73 ± 0.44	6.17 ± 0.23	-2.36 ± 1.21	0.011 ± 0.03
2: p-lys clade 2	216.11 ± 1.75	23.66 ± 0.28	7.07 ± 0.27	-0.11 ± 0.56	0.018 ± 0.03
3: i-lys	155.60 ± 9.89	17.33 ± 1.21	7.29 ± 1.16	1.50 ± 1.27	-0.297 ± 0.04

ANOVA					
*F*_2,30_	153.548	106.883	4.725	3.088	25.517
*P*	< 0.0001	< 0.0001	0.0164	0.0603	< 0.0001

Posthoc					
Tukey-Kramer	1↔2, 1↔3, 2↔3	1↔2, 1↔3, 2↔3	1↔3		1↔3, 2↔3

The comparison focuses on the two clades of the protist-type lysozymes (p-lys) and one clade of the invertebrate-type lysozymes (i-lys), using ANOVA and the Tukey-Kramer posthoc tests performed with the program JMP IN 5.1.2. Significant pairwise posthoc comparisons are indicated by the respective clade numbers. Protein length, molecular weight (MW, to be multiplied by 1000), isoelectric point (pI), charge, and grand average hydropathy are given with the standard error of the mean.

For the *C. elegans *lysozymes the current knowledge on the site of gene expression and the role in immune defence is summarized in Table 8. All genes, for which data is available, appear to be expressed in the intestines. Some are additionally expressed in neurons (*Cel-lys-1*), larval muscles (*Cel-lys-7*), or the pharynx (*Cel-lys-8*). The data on immune function highlights clear differences between the three clades. The most pronounced effect is seen for pathogen-induced gene expression. It was reported for all of the clade 1 protist-type genes. Within this clade, individual genes vary as to their response to different pathogens (Table 8). In contrast, both the clade 2 protist-type and the invertebrate-type genes show considerably fewer cases of pathogen-activation, and at the same time, several cases of pathogen-suppression (Table 8). The above pattern is generally confirmed by the current data on lysozyme regulation through known components of the *C. elegans *immune system (Table 8). The clade 1 protist-type genes generally appear to be under positive control of the immune system. At the same time, they show variation as to the importance of different regulatory factors. In contrast, the other two clades rather appear to be under negative influence of immunity pathways (Table 8).

**Table 8:** Information on the function of the *C. elegans *lysozymes

Clade	Gene	Expression^a^	Immune system		Pathogens		References
					
			Up	Down	Up	Down	
p-lys clade 1	*Cel-lys-1*	I, ILN, HN	SEK-1, NSY-1, TIR-1, DBL-1	DAF-16	BT, EC, EF, PA, PL, SM		[20, 41, 43, 45, 76–80]
	*Cel-lys-2*	I	SEK-1, PMK-1^§^, ELT-2		PA, PL	BT	[43, 45, 76–79]
	*Cel-lys-3*			DAF-16	BT, EC, MN, PA		[19, 41, 43, 78, 79]
	*Cel-lys-7*	I, LM	DAF-16, DBL-1		EF, MN*, SM	PL	[19, 20, 40, 41, 76, 79–81]
	*Cel-lys-8*	I, PB, PG	DBL-1, SMA-2, SEK-1, NSY-1, DAF-16		MN, SM		[19, 20, 40, 42–44, 76, 79, 80]

p-lys clade 2	*Cel-lys-4*	I		SEK-1	EF		[43, 76, 77, 79]
	*Cel-lys-5*	I		PMK-1^§^	EF	PA	[43, 76, 79]
	*Cel-lys-6*	I				PA	[43, 76]
	*Cel-lys-10*		PMK-1^§^		EF, PL		[43, 79]

n.a.	*Cel-lys-9*						

i-lys	*Cel-ilys-1*						
	*Cel-ilys-2*	I			MN, PL	PA	[19, 45, 76, 79]
	*Cel-ilys-3*	I	DAF-16		MN^#^, PL	PA	[19, 40, 45, 77, 79]
	*Cel-ilys-4*			DAF-16	PA		[41, 43]
	*Cel-ilys-5*	I		DAF-16		PA	[41, 43, 77]

The site of expression is given as I, intestines; ILN, the six IL1 and the six IL2 neurons; HN, unidentified head neurons; PB, terminal pharyngeal bulb; PG, pharyngeal gland cells; and LM, L1 muscle cells. Up- or downregulation by immune system components or pathogens is indicated. The regulatory elements of the immune system are denoted with their standard gene names [55]. Pathogens are abbreviated as follows: BT, Cry5 toxin from *Bacillus thuringiensis*; EC, *Erwinia carotovera*; EF, *Enterococcus faecalis*; MN, *Microbacterium nematophilum*; PA, *Pseudomonas aeruginosa*; PL, *Photorhabdus luminescens*; SM, *Serratia marcescens*. ^§ ^*pmk-1 *regulation in a *daf-2 *mutant background. * Increased susceptibility in KO mutants and after RNAi gene silencing. ^# ^Increased susceptibility after RNAi gene silencing.

## Discussion

### Evolution of *Caenorhabditis *lysozymes

*Caenorhabditis *nematodes are among the organisms with the highest number and the most extreme diversity of lysozyme genes. Their lysozymes fall into three distinct clades, one being part of the invertebrate-type and the other two of the evolutionary very distant protist-type lysozymes. Moreover, the *Cel-lys-9 *gene from *C. elegans*, which undoubtedly belongs to the protist-type lysozymes (Fig. 2), shows only limited similarities to the other nematode genes and it may thus represent a class of its own. To date, it is impossible to say whether the invertebrate-type and the protist-type lysozymes evolved from a common ancestor or not. In the latter case, their general similarity as lysozymes would be a consequence of convergent evolution towards a similar function in defence or digestion. Additional data from more basal nematode as well as metazoan taxa (e.g. cnidarians, poriferans, platyhelminths) is required to distinguish between these alternatives.

Some of the *Caenorhabditis *lysozyme genes are found in clusters within the genome, as known for about one fifth of the protein-coding genes of *C. elegans *and apparently characteristic for genes involved in interactions with the environment [26]. Thus, lysozymes may be subject to similar evolutionary dynamics recently described for several of the clustered gene families [27]. These clustered gene families are most likely shaped by concerted molecular evolution. They are characterized by species-specific clades of the gene clusters, the presence of inverted genes that have been proposed to stabilize concerted evolution of clusters over time, and strong purifying selection [27]. However, the inferred evolutionary history of lysozyme clearly contrasts with such patterns. Genes in close genomic proximity do not form species-specific phylogenetic clades. None of the genomic lysozyme clusters contain "stabilizing" genes with inverted orientation in the middle of the cluster. Furthermore, although the majority of genes appears to be subject to purifying selection, we did obtain a strong indication for several episodes of diversifying selection.

We conclude that the lysozymes follow a different evolutionary trajectory. Our analysis reveals three main patterns.

(i) Gene duplication prior to species separation and maintenance of the duplicated genes. This scenario is most evident where lysozyme orthologues are monophyletic and distributed in synteny across genomes in all three taxa, e.g. the protist-type *lys-1*, *lys-2*, and *lys-3 *genes. Other likely cases are the protist-type *lys-6*, *lys-8*, *lys-10*, and the invertebrate-type *ilys-4 *and *ilys-5 *genes, for which corresponding orthologues fall into monophyletic clades. In all these cases, the orthologous genes must have an age of at least three million years, which is the minimum time since the last most common ancestor of the three *Caenorhabditis *species [28]. Their maintenance across time suggests an important conserved biological role for each group of orthologues. In this case, their original divergence after gene duplication may have been favoured by diversifying selection and thus, it may associate with signatures of adaptive sequence evolution. Such a signature is indeed found for the clade 1 protist-type lysozymes (including *lys-1 *to *lys-3*, *lys-8*, and orthologues).

(ii) Recent gene duplication and diversification. Phylogenetic analysis revealed five cases of lineage-specific duplication events (Figs. 4, 7, and 8). One of these cases (*Cre-ilys-4.1 *and *Cre-ilys4.2*) is associated with a significant signature of adaptive sequence evolution, suggesting that diversifying selection favoured lysozyme differentiation upon duplication. The other four cases (*Cre-lys-8.1 *and *Cre-lys-8.2*; *Cbr-lys-6.1 *and *Cbr-lys-6.2*; *Cel-lys-5 *and *Cel-lys-6*; *Cel-lys-7 *and *Cel-lys-8*) appear to be subject to purifying selection. This pattern indicates strong selection for maintenance of gene function after the duplication event.

(iii) Gene duplication prior to species separation followed by differential gene loss. This scenario appears to apply to the *Cel-ilys-1*, *Cel-ilys-2*, *Cel-ilys-3*, and *Cel-lys-4 *genes, which are each present in only one of the species and diverge from internal nodes, some of them along long branches indicative of old evolutionary age. Loss of genes after duplication events in the other Caenorhabditis species may then suggest redundant functions of lysozymes in these taxa. As above under (i), their original diversification may have been driven by diversifying selection. Indeed, two episodes of adaptive sequence evolution were found to associate with these genes (Figs. 4A, 8A).

Phylogenetic inferences can only yield an approximation of the past and thus come with some uncertainty. Considering that the inferred relationships are generally supported by high bootstrap values and that they are based on the maximum likelihood approach, which was shown in the past to be less susceptible to biases (e.g. long-branch attraction) than other tree reconstruction methods [29], our results should provide a realistic image of *Caenorhabditis *lysozyme evolution. Taken together, their lysozyme repertoire is shaped by both ancestral and recent gene duplications. Sequence evolution is to a large extent determined by purifying selection. Yet, it also includes several episodes of diversifying selection, which associate with ancient as well as recent duplications. To our knowledge, similar evolutionary dynamics have not as yet been inferred for the lysozymes from other taxa.

It is worth noting that we did not find an indication for adaptive sequence evolution between the two main protist-type clades (Fig. 6, Table 4). Two explanations are conceivable. On the one hand, differentiation of the two clades was not subject to diversifying selection. On the other hand, diversifying selection was important but could not be detected due to a lack of power of the analysis, which had to be based on a reduced data set including only the conserved sequence regions that could be reliably aligned across the different genes and taxa. At the same time, this specific result (as well as all other cases of comparatively long branches with *d*_*N*_/*d*_*S *_rate ratios below 1) strongly suggests that our analysis is not compromised by a possible saturation of synonymous substitutions along long branches, which could have led to underestimated *d*_*S *_rates and thus artificially high *d*_*N*_/*d*_*S *_rate ratios. It is also worth noting that only a single alignment site was inferred to be under positive selection in our analyses. This is unusual because in most immunity gene data sets associated with adaptive sequence evolution a larger number of positively selected sites is identified, e.g. in MHC class I receptors [30,31]. A possible reason is that the different evolutionary lineages vary as to the position of the positively selected sites or that only few lineages are subject to positive selection on specific sites. In both cases, the method employed would hinder detection of these positively selected sites because it assumes the same pattern of selection across all lineages [25]. We did not attempt to perform an analysis, in which *d*_*N*_/*d*_*S *_ratios are allowed to vary simultaneously across sites and lineages, because these types of analyses may be liable to higher error rates [32,33]. The single site, which we identified to be under positive selection, is thus predicted to be of main – albeit currently unknown – functional importance.

### Functional diversification

Gene duplications are likely to be one of the main sources of evolutionary innovation [16]. The duplicated genes may acquire new functions (neo-functionalisation) or they may partition the multiple functions of the ancestral gene (sub-functionalisation) [17]. The relevance of either alternative as well as additional scenarios is a topic of intense current debate [34-38]. Importantly, in all cases the genetic diversity of duplicates is predicted to translate into functional diversity. Such a pattern is found in the ruminantia, which possess at least five different lysozyme types: the stomach, tracheal, intestinal, kidney, and milk lysozymes [10]. The first type is involved in digestion, whereas the others may function as antibacterial enzymes in immunity [10]. A similar pattern is observed for the at least eleven different *Drosophila *lysozymes. Most of them have a digestive role and show specialisation as to their time and site of expression [5,14]. A recent study additionally suggested an anti-fungal immune function for some of the genes (Lys B, C, D, E and CG16756) [39]. A further example includes the nine lysozymes of the mosquito *Anopheles gambiae*, which vary as to their role in immunity and digestion and also as to their time and location of expression [15].

The *Caenorhabditis *lysozymes show clear signatures of functional diversification. Pronounced differences between the three main clades and also within each of the clades are observed for molecular characteristics of the genes, their pathogen-induced expression, and also their regulation by the immune system. Based on the current data, it appears that the protist-type clade 1 lysozymes play an important role in immunity: They are all induced upon pathogen exposure. Most of them are under positive control of immunity pathways, including components of the insulin-like signalling cascade (DAF-16) [40-42], the p38 mitogen-activated protein kinase (MAPK) pathway (SEK-1 and PMK-1) [43], the TGF-β pathway (DBL-1, SMA-2) [44], or the GATA transcription factor ELT-2 [45]. Most interestingly, the different genes from this clade vary in their response to pathogens and immunity pathways. This variation may contribute to high immune specificity, as has recently been identified phenomenologically for invertebrates [46-48] and which is consistent with highly specific *C. elegans*-pathogen interactions [49]. Although the underlying molecular mechanisms are currently unknown, they are likely to be based on the genetic diversification of pathogen recognition receptors and/or immune effectors such as the lysozymes [21,50,51]. They may also include the synergistic interaction between different components of the immune system [51], as generally known for lysozymes and antimicrobial peptides [5,7,52,53]. In *C. elegans*, the immune function has been tested for two genes of the clade 1 protist-type lysozymes. Overexpression of *Cel-lys-1 *enhances resistance against *S. marcescens *[20], whereas silencing of *Cel-lys-7 *increases susceptibility to *M. nematophilum *[19]. The importance of lysozyme diversification for immunity in general and also for immune specificity clearly warrants further investigation.

The role of the invertebrate-type and also the clade 2 protist-type lysozymes is as yet unclear. The only exception may be *Cel-ilys-3*. Its silencing enhances susceptibility to *M. nematophilum *[19]. In the same study, no effect was observed after *Cel-ilys-2 *knock-down [19]. In general, both invertebrate-type and clade 2 protist-type lysozymes are less often activated by pathogens than the clade 1 protist-type lysozymes. At the same time, several of the genes are downregulated by pathogens and by known immunity pathways. The latter observation may suggest that their main function somehow interferes with the immune response. A similar finding was made for some of the digestive lysozymes from *D. melanogaster*, which are also downregulated upon immune challenge [5]. This particular similarity may indicate that the primary function of these nematode lysozymes is also digestion. The information on their molecular characteristics (e.g. isoelectric point) or the localization of gene expression is consistent with a role in both immunity and digestion. Unfortunately, the nematode's intestines are the main location for bacterial digestion and at the same time immune defence against pathogens that are easily taken up during feeding [54]. Therefore, lysozymes are expected to have similar characteristics (e.g. regarding pH optimum) even if they vary in function. Future analyses should thus be performed with either exclusive food bacteria or exclusive pathogens, in order to distinguish between the alternative functions.

## Conclusion

Our study provides an evolutionary framework for understanding lysozyme diversification in *Caenorhabditis *nematodes. The comprehensive lysozyme repertoire falls into three distinct clades and it is shaped by both purifying selection and several episodes of adaptive sequence evolution. The genetic diversification appears to translate into functional differentiation. The information obtained should prove useful as a primer for future analysis of lysozyme function in digestion and immunity. The *Caenorhabditis *lysozymes may further serve as an example of the importance of evolution by gene duplication in invertebrate immune systems.

## Methods

### Sequence alignments

For the three considered *Caenorhabditis *species, protein and DNA sequences of annotated genes with similarities to known lysozymes were obtained from wormbase [55]. Three main alignments were generated (alignments 1, 2, and 4; see below). The first one of these, alignment 1, served to infer the general phylogenetic relationship of the *C. elegans *lysozymes to those from other taxa. We specifically considered taxa, which were included in similar lysozyme phylogenetic analyses in the past [8,56,57], thus allowing comparison between our results and those from previous studies. The alignment was produced with the hierarchical method, implemented in the programme CLUSTALW [58] and using the default settings. The resulting alignment contained substantial sequence variation. Moreover, variations of the programme settings (gap open, gap extension, and gap distance penalties) resulted in differences among generated alignments. Therefore, positional homology across alignment 1 may not be entirely reliable. Since it is based on the hierarchical alignment method (i.e. similar sequences are aligned first, followed by subsequent addition of less similar sequences), it should still be informative as to the general phylogenetic position of the Caenorhabditis lysozymes in comparison to those from other taxa.

The more detailed analysis of Caenorhabditis lysozyme evolution was based on the main alignments 2 and 4. Six additional alignments were extracted from these two alignments (see below, alignments 3, 5–9). In particular, alignments 2 and 3 served to analyse the invertebrate-type lysozymes. The overall phylogeny of the protist-type lysozymes from nematodes and one outgroup taxon was examined with alignment 4. Five additional alignments were extracted from alignment 4 for the detailed analysis of lysozyme evolution (alignments 5–9). Here, we excluded highly variable sequence regions from alignments, if these could not be recovered in identical form under alternative settings of the alignment programme (only relevant for alignments 5–7), in order to ensure a high likelihood of positional homology and thus a reduced risk of homoplasy. Alignments 4–9 are subsets of each other with identical position of indels as indicated in Fig. 5.

(i) Alignment 1 included protein sequences of all lysozyme genes from *C. elegans *but none of the other *Caenorhabditis *species. The *C. elegans *genes were combined with lysozymes from various taxa that have previously been considered in similar phylogenetic analyses [8,56,57], including the chicken-type lysozyme from chicken (*Gallus gallus*, accession number CAA23711), one of the chicken-type lysozymes from mice (*Mus musculus*, AAA39473), the goose-type lysozyme from chicken (NP_001001470), two chicken-type and one invertebrate-type lysozymes from *D. melanogaster *(NP_523882 [previously AAF47448], NP_476828 [previously AAF47452], and CAA21317), one chicken-type and one invertebrate-type lysozyme from the mosquito *Anopheles gambiae *(AAC47326, AAT51799), the invertebrate-type lysozymes from the cestode *Tapes japonica *(BAB33389), the molluscs *Mytilus edulis *(AAN16207), *Chlamys islandica *(CAB63451), and *Calyptogena *sp. 1 (AF334666), the leech *Hirudo medicinalis *(AAA96144) and the sea star *Asterias rubens *(AAR29291), and four protist-type lysozymes from *Dictyestelium discoideum *(XP_644284, AAM08434, AAB06786, XP_643993), two from *Entamoeba histolytica *(AAC67235, Q27650), and one from *Tetrahymena thermophila *(XP_001008528), and one lysozyme from a T4 entobacteria phage (1LYD).

(ii) Alignment 2 contained protein sequences of all invertebrate-type lysozymes from the three *Caenorhabditis *species (Fig. 3).

(iii) Alignment 3 is the DNA version of alignment 2.

(iv) Alignment 4 has all protein sequences of the *Caenorhabditis *protist-type lysozymes and also one from *D. discoideum *(XP_644284).

(v) Alignment 5 is the modified DNA version of alignment 4. Here, we excluded the taxon *D. discoideum *and additionally one 5' and one 3' end region, which could not be aligned reliably at the DNA sequence level. The excluded region at the 5' end corresponds to positions 1 to 149 and that at the 3' end to positions 301 to 345 of the protein sequence alignment (see Fig. 5).

(vi) Alignment 6 represents a subset of alignment 4. It contains the *Caenorhabditis *protein sequences of the clade 1 protist-type lysozymes, whereby we excluded a fragment at the 5' end (positions 1 to 67; Fig. 5), another fragment towards the 3' end (positions 314 to 331; Fig. 5), and a small region at the 3' end (positions 339 to 345; Fig. 5).

(vii) Alignment 7 is the DNA version of alignment 6.

(viii) Alignment 8 is again a subset of alignment 4. It includes all complete *Caenorhabditis *protein sequences of the clade 2 protist-type lysozymes (Fig. 5).

(ix) Alignment 9 is the DNA version of alignment 8.

### Sequence characteristics

General properties of the different lysozymes were inferred with the help of the ProtParam tool of the ExPASy server [59,60], including protein length, molecular weight, isoelectric point (pI), charge, and also the grand average of hydrophobicity. Differences in these traits between lysozyme clades were assessed with an analysis of variance (ANOVA) and posthoc Tukey-Kramer comparisons, using the program JMP IN 5.1.2 (SAS Institute Inc.). The presence and position of a signal peptide was inferred with the SIGNALP 3.0 server [61]. Further information on the genomic location, the function and also regulation of the *C. elegans *lysozymes were taken from wormbase [55] and the current literature.

### Phylogenetic tree inference

Phylogenies were reconstructed using the maximum likelihood (ML) optimality criterium [23]. For protein sequence alignments, the optimal substitution model was first inferred using the program ProtTest version 1.3 [62] and the Akaike information criterion, following the recommended approach [63,64]. The optimal substitution model was employed for a heuristic tree search with the help of the program PhyML [65,66] using default settings. The robustness of the inferred tree topology was evaluated via non-parametric bootstrapping [67] based on 200 replicate data sets.

For DNA sequence alignments, the optimal substitution model was found using the same strategy as above and as implemented in the program ModelTest version 3.7 [63,64,68]. The phylogenetic tree was then inferred with the help of the ML option of the program PAUP* 4.0b10 [69], using the optimal substitution model, a heuristic tree search based on branch-swapping by tree bisection and reconnection (TBR), the random addition of sequences, which was repeated ten times, and otherwise default settings. The robustness of the tree topology was assessed with non-parametric bootstrapping using 500 replicates.

### Analysis of adaptive sequence evolution

The presence of positive selection (i.e. a *d*_*N*_/*d*_*S *_rate ratio larger than 1) along branches of the different tree topologies or along the sequence alignments was assessed using the ML approach implemented in the program CODEML of the PAML package version 3.15 [70]. DNA sequences of the coding regions were used as input data files (alignments 3, 5, 7, and 9) and the inferred unrooted ML tree topologies as input tree files. Positive selection along sequence alignments was inferred following recommendations [71,72]. In particular, likelihood ratio tests (LRT) were used to compare the NS sites model 8 (8 rate categories across sequences, one of which was allowed to have a *d*_*N*_/*d*_*S *_rate ratio larger than 1) with either NS sites model 7 (8 rate categories, none above 1) or NS sites model 8a (8 rate categories, whereby one was set to exactly 1). The significance of the comparison between models 8 and 8a was assessed by dividing the inferred LRT probability by 2, as recommended previously [72]. If both comparisons were significant, then individual sites under positive selection were identified using the Bayes empirical Bayes method [71].

The presence of positive selection along branches was assessed using two approaches. On the one hand, we compared a model, in which all branches were forced to have the same *d*_*N*_/*d*_*S *_rate ratio (1-ratio model), with a model, in which one branch was allowed to differ whereas all others were forced to be identical (2-ratio model). Using this approach, we tested each individual branch of a given tree topology. The significance of the comparison was evaluated with a likelihood ratio test [13]. We corrected for multiple testing by adjusting the significance level according to Bonferroni [73] and the false discovery rate (FDR) [74,75]. If a particular comparison was significant and if the individual branch, which was allowed to differ, had a *d*_*N*_/*d*_*S *_rate ratio larger than 1, then this was taken as an indication for positive selection along this branch.

On the other hand, we assessed the significance of positive selection along branches using non-parametric bootstrapping. In particular, we first used the free-ratio model, in which all branches were allowed to vary, in order to calculate *d*_*N*_/*d*_*S *_rate ratios for each individual branch of the topology. The significance of a value either above or below 1 was tested by repeating the calculations on 100 bootstrapped data sets. Non-parametric bootstrapping of the data was performed in consideration of the coding structure of the genes using the program CODEML of the PAML package [70]. If a specific branch had a *d*_*N*_/*d*_*S *_rate ratio larger 1 and bootstrap support of more than 50, then this was taken as an indication of positive selection.

## Authors' contributions

HS conceived the study and performed the phylogenetic analyses, HS and CB together collected data on sequence characteristics and wrote the manuscript.

## Supplementary Material

Additional file 1 **Alignment of the *Caenorhabditis *protist-type lysozyme amino acid sequences**. The additional fileshows the complete alignment. The top quarter of the alignment is given in Figure 5. In both cases, black lines at the beginning of the alignment denote the inferred signal peptides. Alignment 4 (see methods and results) includes all taxa and the entire protein sequences. Vertical black lines with arrows below the alignment indicate the regions used for specific DNA sequence analysis of all protist-type lysozymes (alignment 5). Vertical black lines with arrows above the alignment indicate those regions analyzed for the clade 1 lysozymes (alignments 6 and 7 for protein and DNA sequences, respectively). Clade 2 lysozyme analysis was based on complete sequences (alignments 8 and 9 for protein and DNA sequences, respectively). Note that all alignments are subsets of alignment 4, i.e. the position of indels is identical. The red box and arrow indicate the sequence position, which was inferred to be under positive selection for the clade 1 lysozymes. Click here for file
