# Supplementary figures and images for: Diversification and adaptive sequence evolution of Caenorhabditis lysozymes (Nematoda: Rhabditidae)
Source: BMC Evol Biol. 2008 Apr 19;8:114. doi: 10.1186/1471-2148-8-114 (PMC2383907; doi:10.1186/1471-2148-8-114)

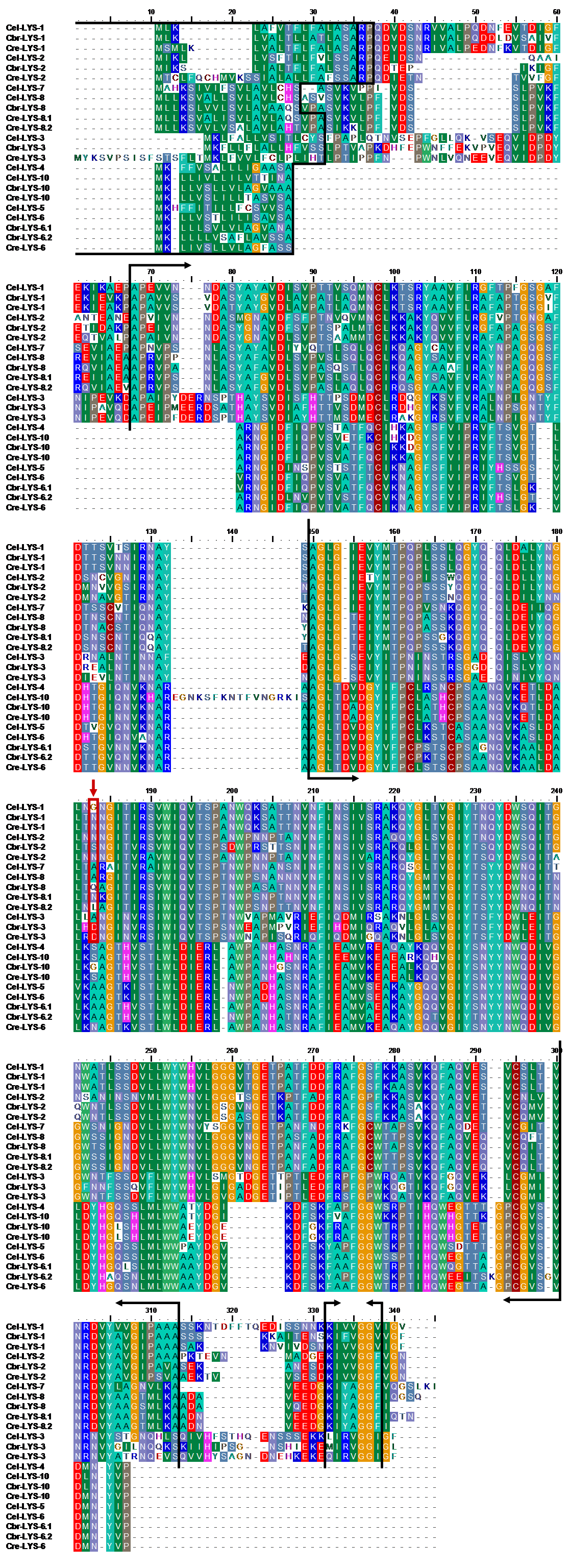

Supplement: Additional file 1 — Alignment of the Caenorhabditis protist-type lysozyme amino acid sequences. The additional fileshows the complete alignment. The top quarter of the alignment is given in Figure 5. In both cases, black lines at the beginning of the alignment denote the inferred signal peptides. Alignment 4 (see methods and results) includes all taxa and the entire protein sequences. Vertical black lines with arrows below the alignment indicate the regions used for specific DNA sequence analysis of all protist-type lysozymes (alignment 5). Vertical black lines with arrows above the alignment indicate those regions analyzed for the clade 1 lysozymes (alignments 6 and 7 for protein and DNA sequences, respectively). Clade 2 lysozyme analysis was based on complete sequences (alignments 8 and 9 for protein and DNA sequences, respectively). Note that all alignments are subsets of alignment 4, i.e. the position of indels is identical. The red box and arrow indicate the sequence position, which was inferred to be under positive selection for the clade 1 lysozymes. [file 1471-2148-8-114-S1.TIFF]
